# Supplementary material for: Synergistic Interaction between Copper and Nitrogen-Uptake, Translocation, and Distribution in Rice Plant
Source: Plants (Basel). 2022 Oct 4;11(19):2612. doi: 10.3390/plants11192612 (PMC9572941; doi:10.3390/plants11192612)
Supplement: Supplementary file 1 [file plants-11-02612-s001.zip › plants-1939827-supplementary.pdf]

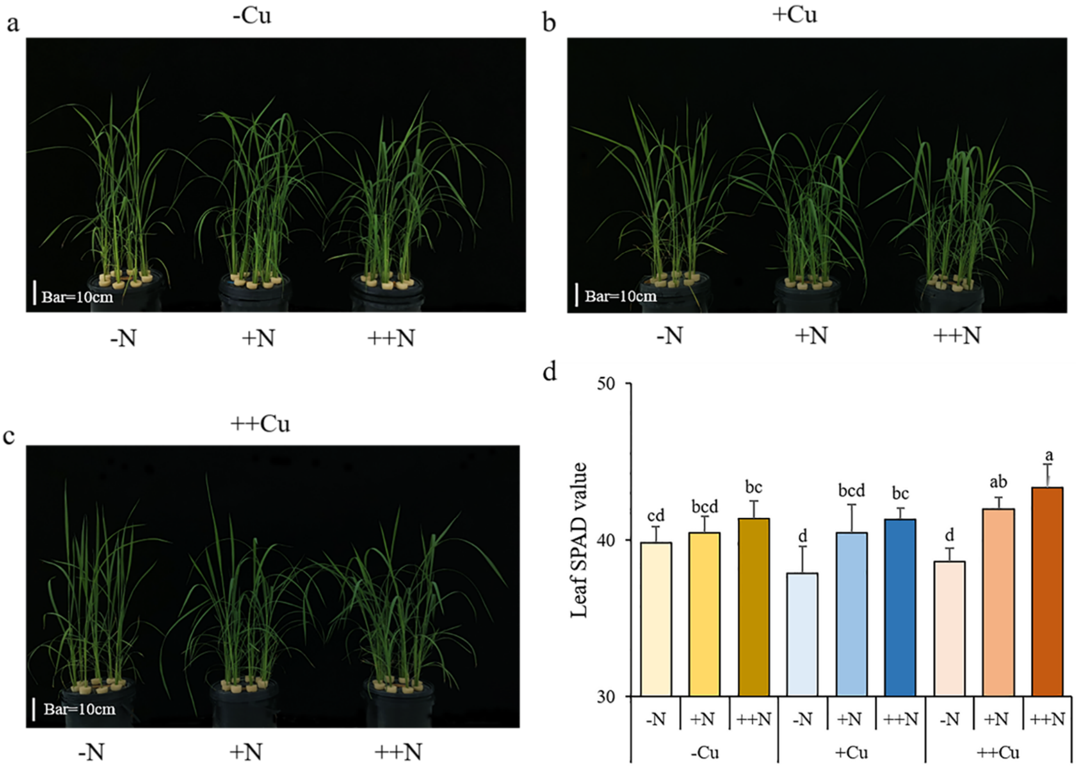

Figure S1. Growth phenotype (**a**–**c**) and leaf SPAD value (**d**) of rice plant grown in solutions with different combinations of Cu (-Cu, 0 μM; +Cu, 0.15 μM; ++Cu, 1.5 μM) and N (-N, 0.288 mM; +N, 2.88 mM; ++N, 14.4 mM) supply for three weeks. Data are means ±SD of three biological replicates. Different letters indicate significant difference at $p < 0.05$ by Tukey's test.

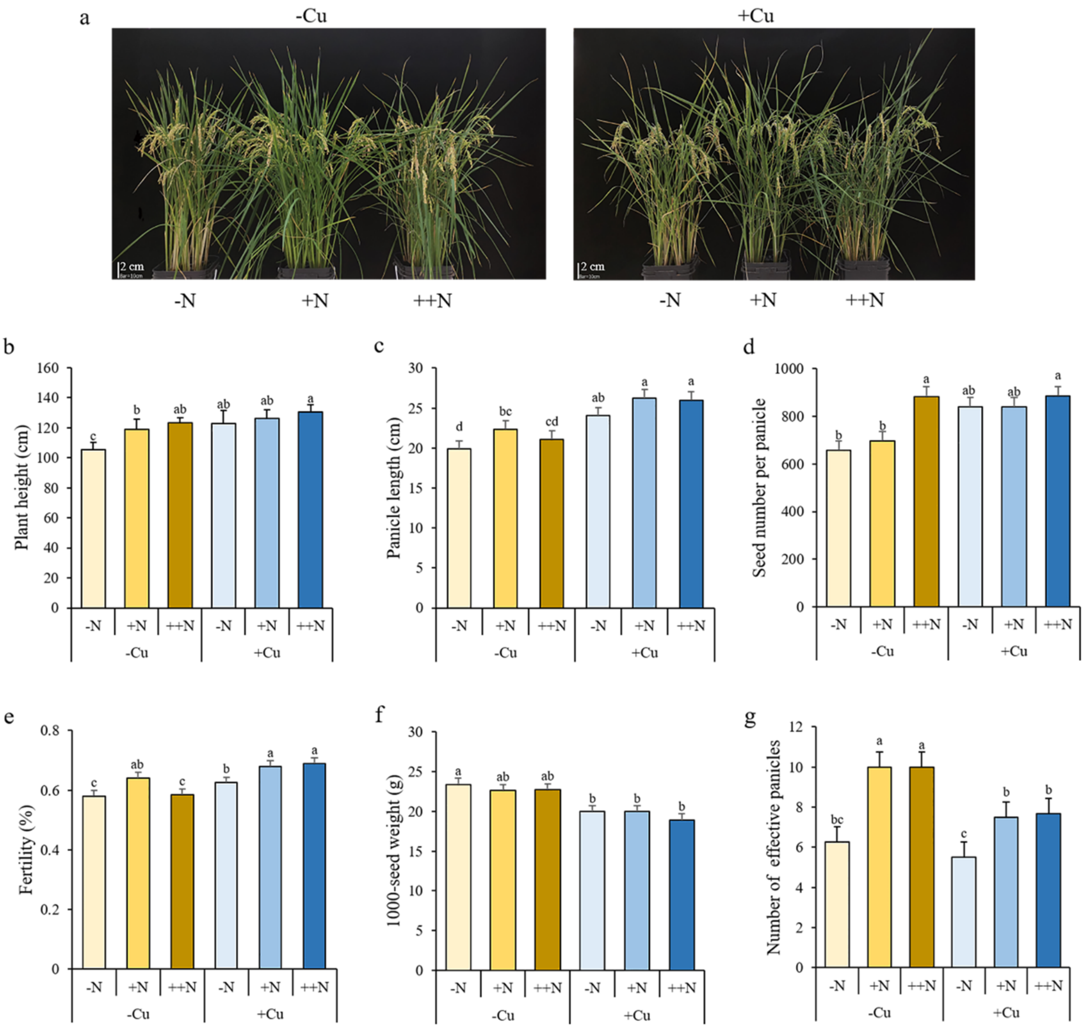

Figure S2. Growth phenotype (**a**) and yield components (**b**–**g**; **b**, plant height; **c**, panicle length; d, seed number per panicle; e, fertility; f, 1000-seed weight; g, number of effective panicles) of rice plant grown in soil with different combinations of Cu (-Cu, 0 mg Cu kg^{-1} soil; +Cu, 50 mg Cu kg^{-1} soil) and N (-N, 0 g N kg^{-1} soil; +N, 0.2 g N kg^{-1} soil; ++N, 0.4 g N kg^{-1} soil) supply at mature stage. Data are means ±SD of three biological replicates. Different letters indicate significant difference at $p < 0.05$ by Tukey's test.

Table S1. Primers used in qRT-PCR.

Gene Name	Primer Name	Primer Sequence (5′ to 3′)
OsUbiquitin	OsUbiquitin-F	GACGGACGCACCCTGGCTGA
	OsUbiquitin-R	TGCTGCCAATTACCATATACC
OsAMT1.1	OsAMT1.1-RT-F	ACCAATCCAGAACGAACGAC
	OsAMT1.1-RT-R	AGCCCAGCAAAAGTGAAAGA
OsAMT1.2	OsAMT1.2-RT-F	CTTCATCGGGAAGCAGTTCT
	OsAMT1.2-RT-R	TGAGGAAGGCGGAGTAGATG
OsAMT1.3	OsAMT1.3-RT-F	GCGTGGGGGATCATCTTCAC
	OsAMT1.3-RT-R	GAACCTGTGCAGCACGTAGA
OsAMT2.1	OsAMT2.1-RT-F	GCGTTCGTGATCGCGTGGA
	OsAMT2.1-RT-R	TAGAGCTGGATGGTGACGC
OsAMT2.3	OsAMT2.3-RT-F	TCACCATTCAGTTGTAGAAGACGG
	OsAMT2.3-RT-R	CTACACCAGCATTACATCACCGAA
OsAMT3.1	OsAMT3.1-RT-F	CTTCATCATCTGCTGGAACGTG
	OsAMT3.1-RT-R	TTGTTGTTGTGGTGCGTGTC
OsAMT3.2	OsAMT3.2-RT-F	CTCACCTTCTCCTACACCGTC
	OsAMT3.2-RT-R	ACCCCATCCATAGTAACCCTG
OsAMT3.3	OsAMT3.3-RT-F	CGAGCATCACCATCATCATC
	OsAMT3.3-RT-R	ATGACACCCCACTGGAAGAG
OsNRT1.1A	OsNRT1.1A-RT-F	CCCACACCAAGCAATTCAGG
	OsNRT1.1A-RT-R	GTCTTCACCTCCTCCACGTC
OsNRT1.1B	OsNRT1.1B-RT-F	CAACCTGGTGCCGTACATGA
	OsNRT1.1B-RT-R	TCGTGAGTATCATCACGCCG
OsNRT2.1	OsNRT2.1-RT-F	CACGGTGCAAGTCTCAAG
	OsNRT2.1-RT-R	GGTATAAATGCCTCTCCC
OsNRT2.2	OsNRT2.2-RT-F	CGGAGCACGCCTAATTAAGAG
	OsNRT2.2-RT-R	CTCCATGACGACATACTCTAGATA
OsNRT2.3a	OsNRT2.3a-RT-F	CGCTGCTGCCGCTCATCCG
	OsNRT2.3a-RT-R	CCGTGCCCATGGCCAGAC
OsNRT2.4	OsNRT2.4-RT-F	AAAGGTCGCTGGGCGTGGTG
	OsNRT2.4-RT-R	CCTGGACCCGCTGAAGAAGAG
OsNR1	OsNR1-RT-F	TACCAGGTCATCCAGTCGGT
	OsNR1-RT-R	GTACTTCCACCCTTCCTCCG
OsNR2	OsNR2-RT-F	CAAGGTGTGGTACGTGGTGA
	OsNR2-RT-R	GCAGGACTTGTCGAGGTCAT
OsNiR1	OsNiR1-RT-F	CTGCCTCACCAAGGACAG
	OsNiR1-RT-R	TTCCTACTCCTCGTCCTCCT
OsNiR2	OsNiR2-RT-F	GAACGAGGAGTAGGAGCACA
	OsNiR2-RT-R	GGGCTACAAGATCAAACCAA
OsGS1.1	OsGS1.1-RT-F	TGGGTTGCTCGCTACATTCT
	OsGS1.1-RT-R	GCTTGAGCTTCTCAATGGCG
OsGS1.2	OsGS1.2-RT-F	TGCTGACCAAGTGTGGGTTG
	OsGS1.2-RT-R	GAGCTTGTCGATCGCCTTCT
OsGS2	OsGS2-RT-F	TTTCTCATGGGGTGTGGCAA
	OsGS2-RT-R	GGGCCAACTTCTTAGCAGCA
OsFd-GOGAT	OsFd-GOGAT1-RT-F	CGGGTGGCCTTGCCTATATT
	OsFd-GOGAT1-RT-R	ACCAGTTGCCAGAAGAGTGG
OsNADH-GOGAT	OsNADH-GOGAT2-RT-F	CCTGTCGAAGGATGATGAAGGTGA A
	OsNADH-GOGAT2-RT-F	TGCATGGCCCTACTATCTTCGCATCA
OsCOPT1	OsCOPT1-RT-F	TTGCAGCCTGGAGAAAACAC

	OsCOPT1-RT-R	TCCACTTCACAGTCCGTCTC
OsCOPT3	OsCOPT3F	GCTCGCCGTCATGTCCTTCA
	OsCOPT3R	TCTGACGATGGATGGAGAGA
OsCOPT5	OsCOPT5-RT-F	CGCGATCCTCTTCATGTTCG
	OsCOPT5-RT-R	CGCGAGCATGATGAGGTATG
OsCOPT7	OsCOPT7F	CCGCCTTCTACCAGTACCTC
	OsCOPT7R	CATGAGGAGGTAGCCCAGG
OsHMA5	OsHMA5-RT-F	GAGAGCTTCTGTCGCATTGG
	OsHMA5-RT-R	TGCCATCCACTTTGAGGTCT
OsHMA9	OsHMA9-RT-F	TGCTATCTCTCAGCCAAAGG
	OsHMA9-RT-R	CTAGTGAGGTTGCTAAGGCA
OsYSL16	OsYSL16-RT-F	GTGGATTCGGGTCGTTCTTG
	OsYSL16-RT-R	GTCGATGACCAGCACCTTTC
OsATX1	OsATX1-RT-F	TTACACCAGATGCCGTTCTTC
	OsATX1-RT-R	AGCAGCAGTAGCTTCAACAG
